# Supplementary material for: A quantitative analysis of various patterns applied in lattice light sheet microscopy
Source: Nat Commun. 2022 Aug 8;13:4607. doi: 10.1038/s41467-022-32341-w (PMC9360440; doi:10.1038/s41467-022-32341-w)
Supplement: Supplementary file 1 — Supplementary Information [file 41467_2022_32341_MOESM1_ESM.pdf]

Supplementary Material for

A quantitative analysis of various patterns applied in lattice light sheet microscopy

Yu Shi[1], Timothy A. Daugird[2], Wesley R. Legant[1,2]*

1. Joint Department of Biomedical Engineering, University of North Carolina at Chapel Hill, North Carolina State University; Chapel Hill, NC, USA, 27599

2. Department of Pharmacology, University of North Carolina at Chapel Hill; Chapel Hill, NC, USA, 27599

This material includes:

- **Supplementary Tables 1-3**
- **Supplementary Figures 1-24**

(a)

	Propagation length based on optical sectioning (λ)	Propagation length based on fwhm propagation profile(λ)
Gaussian NA 0.21	21.3	22.6
MB Square NA 0.35/0.25	22.2	21.3
Hex NA 0.46/0.36	26.3	22.7
Flat Top NA 0.26	20.2	25.2

(b)

	Propagation length based on optical sectioning (λ)	Propagation length based on fwhm propagation profile(λ
Gaussian NA 0.21	21.3	22.6
MB Square NA 0.31/0.16	18.6	22.9
MB Square NA 0.35/0.25	22.2	21.3
MB Square NA 0.69/0.64	15.0	17.5

(c)

	Propagation length based on optical sectioning (λ)	Propagation length based on fwhm propagation profile(λ
Gaussian NA 0.21	21.3	22.6
Hex NA 0.35/0.2	25.8	22.4
Hex NA 0.46/0.36	26.3	22.7
Hex NA 0.63/0.57	28.4	20.1

Supplementary Table 1: (a) Comparison of Gaussian, Hex, MB square lattice and flat top beams' propagation length based on the optical sectioning doubling distance (left) and FWHM of the intensity profile along the propagation direction (right). (b) Similar table for comparison of different MB square lattice (d) similar table for comparison of different hexagonal lattice

(a)

Beam Type	Overall PSF axial FWHM (λ)		Kz OTF cutoff frequency (fraction of $\frac{4\pi}{\lambda}$)		Optical Sectioning (λ)	
	Center	FWHM	Center	FWHM	Center	FWHM
GS NA 0.21/0	1.83	2.43	0.35	0.24	0.84	1.84
MB Square NA 0.35/0.25	1.62	1.60	0.36	0.32	1.87	3.90
Hex NA 0.46/0.36	1.18	1.27	0.41	0.38	3.42	6.37
Flat Top NA 0.25/0	1.84	2.02	0.34	0.28	0.90	2.32

(b)

Beam Type	Overall PSF axial FWHM (λ)		Kz OTF cutoff frequency (fraction of $\frac{4\pi}{\lambda}$)		Optical Sectioning (λ)	
	Center	FWHM	Center	FWHM	Center	FWHM
GS NA 0.21/0	1.83	2.43	0.35	0.24	0.84	1.84
MB Square ΔNA≈0.15 NA 0.31/0.16	1.61	1.74	0.36	0.30	1.38	3.31
MB Square ΔNA≈0.1 NA 0.35/0.25	1.62	1.60	0.36	0.32	1.87	3.90
MB Square ΔNA≈0.05 NA 0.69/0.64	1.87	2.02	0.58	0.51	1.59	4.11

(c)

Beam Type	Overall PSF axial FWHM (λ)		Kz OTF cutoff frequency (fraction of $\frac{4\pi}{\lambda}$)		Optical Sectioning (λ)	
	Center	FWHM	Center	FWHM	Center	FWHM
GS NA 0.21/0	1.83	2.43	0.35	0.24	0.84	1.84
Hex ΔNA≈0.15 NA 0.35/0.2	1.46	1.58	0.35	0.31	2.53	4.76
Hex ΔNA≈0.1 NA 0.46/0.36	1.18	1.27	0.41	0.38	3.42	6.37
Hex ΔNA≈0.05 NA 0.63/0.57	0.85	0.92	0.54	0.51	5.77	10.04

Supplementary Table 2: (a) Characterization of Gaussian, Hex, MB square lattice and flat top beams with the same propagation length of 20 µm (assuming λ = 560 nm and refractive index n = 1.3). Overall PSF FWHM is calculated based on the intensity profile at x = 0 for the overall PSF. OTF cut-off frequency is calculated based on the frequency at which the OTF strength drops to 0.1 % of the value at k_z = 0. The unit of cut-off frequency is a fraction of 4π/λ as 4π/λ is the theoretically maximum OTF support. Optical sectioning is calculated based on the half width where 63 % of the cumulated intensity falls within. (b) Similar table for comparison of different MB square lattice light sheets with the same propagation length of 20 µm. (c) Similar table for comparison of different hexagonal lattice light sheets with the same propagation length of 20 µm.

(a)

Same Integral

	**High Intensity**		**Low Intensity**	
	Estimated total sample power (μW)	Estimated average intensity at sample (μW/μm²)	Estimated total sample power (μW)	Estimated average intensity at sample (μW/μm²)
Gaussian	120	0.165	44.3	0.060
MB Square	120	0.091	44.3	0.034
Hex NA	120	0.094	44.3	0.034

(b)

Same Peak

	**High Intensity**		**Low Intensity**	
	Estimated total sample power (μW)	Estimated average intensity at sample (μW/μm²)	Estimated total sample power (μW)	Estimated average intensity at sample (μW/μm²)
Gaussian	75.9	0.103	28.2	0.038
MB Square	120	0.091	44.3	0.034
Hex NA	144	0.113	53.4	0.042

Supplementary Table 3: (a) Estimated power at the sample plane for Gaussian, MB square lattice and hexagonal beams with the same integrated energy over 10 μm range. The total power at the sample is estimated based on the measured power at the back pupil plane of the excitation objective and the transmission efficiency of the excitation objective. The intensity is estimated by dividing the total power at the sample with the cross-section area of the beam within which contains 90 % of the total power. (b) similar table as (a) for the three beams with the same peak intensity.

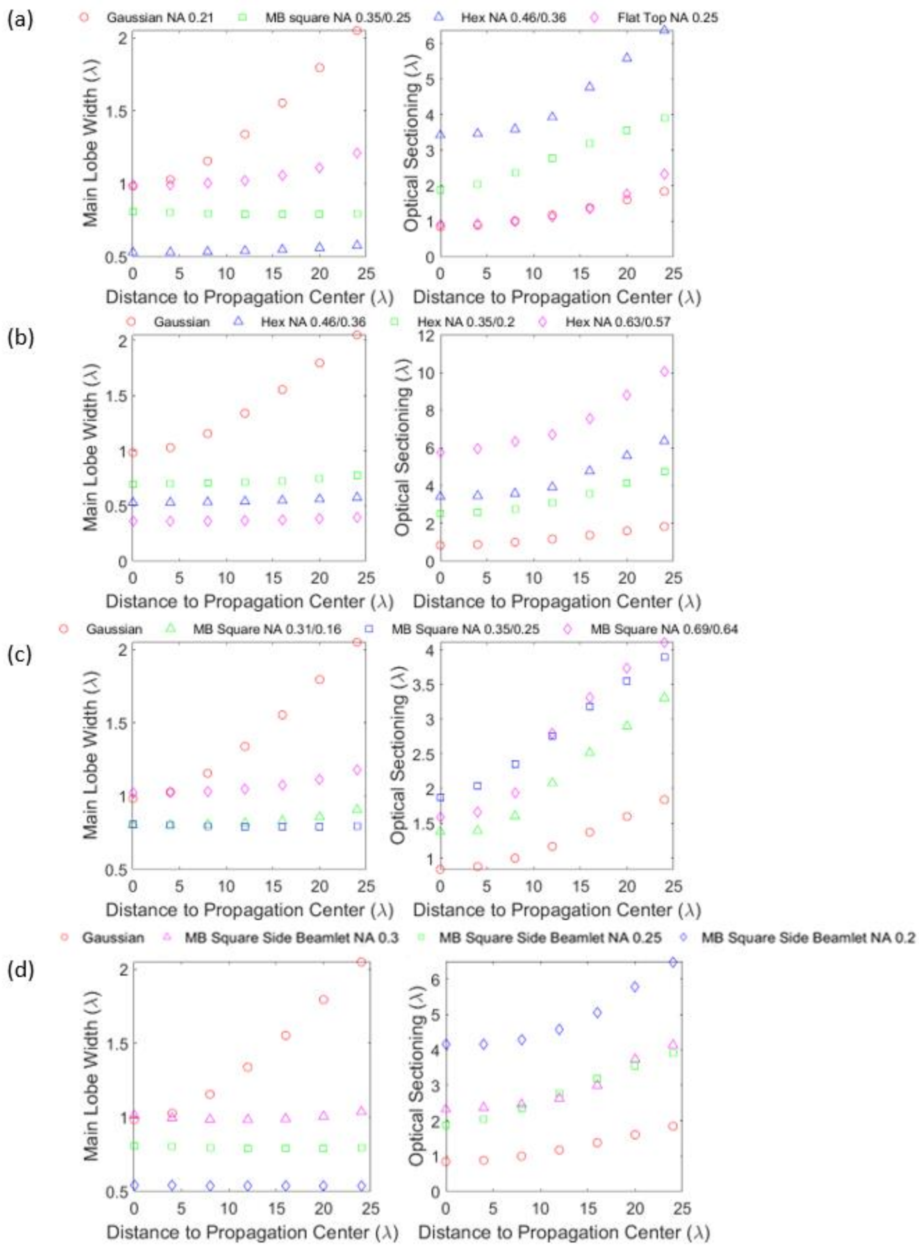

Figure SI1: Beam characterizations following similar metrics to Remacha et al. (a) Main lobe width (left) and optical sectioning (right) for Gaussian, Hex, MB square lattice and flat top with the same propagation length of 20 µm. Main lobe width is defined by the width where the intensity in the excitation PSF along the axial direction (z) drops to 63% of the center. Optical sectioning is defined as the half width where 63 % of the cumulated intensity falls within, and the relative optical sectioning is the optical sectioning compared to when the beam is at the propagation center. (b) Similar to (a) for different Hex beams with the same propagation length of 20 µm. (c) Similar to (a) for different MB square lattice with the same propagation length of 20 µm. (d) Similar to (a) for different MB square lattice with the same min and max NA but different spacing.

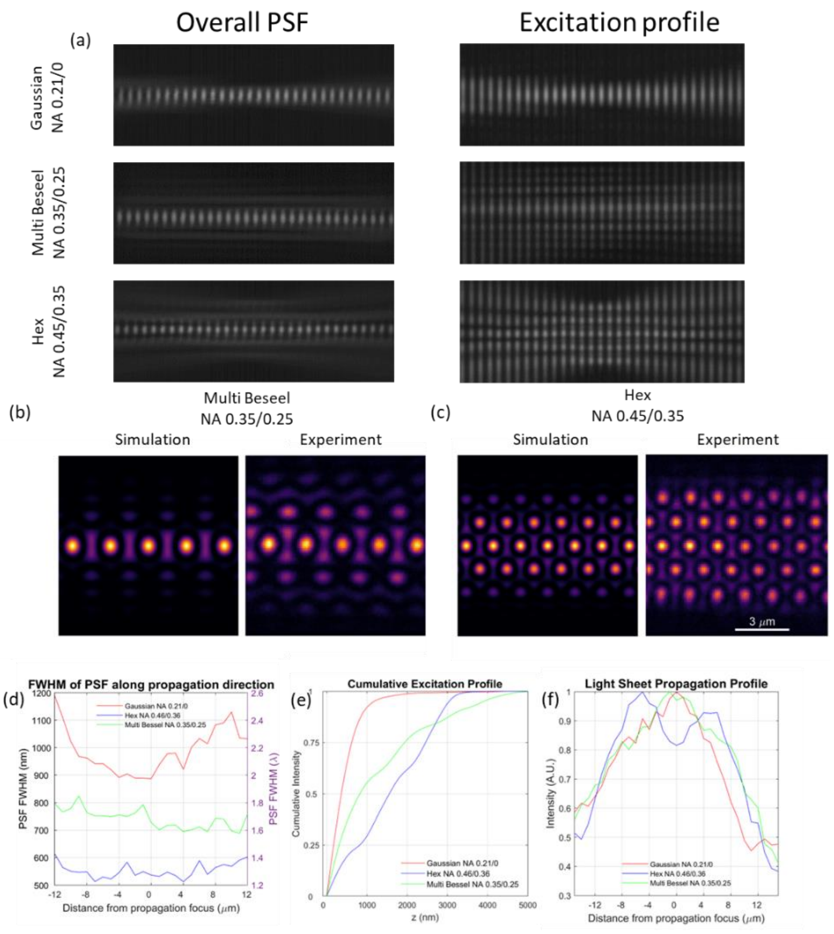

(g)

Beam Type	Overall PSF axial FWHM (λ/nm)		Propagation Length (μm)
	Center	FWHM	
GS NA 0.21/0	1.95/887	2.49/1075	16.8
MB Square NA 0.35/0.25	1.69/728	1.71/738	18.0
Hex NA 0.46/0.36	1.24/537	1.30/559	20.0

Figure SI2: Experimental measurements of single bead with the three beams in Fig 1. (a) Experimental measurement of the overall PSF (left column, corresponding to Fig 1(f) and Fig 1(g)) and excitation profile (right column, corresponding to Fig 1(b)) at different locations along the propagation direction. (b) Excitation profile at the beam focus for MB square lattice for simulation (left) and experiments (right). (c) Excitation profile at the beam focus for hexagonal lattice for simulation (left) and experiments (right) (d) Axial FWHM of the overall PSF along the propagation direction. (e) Cumulative intensity in the excitation profile along the axial direction (corresponding to Fig 1 (e)). (f) Line cut of excitation intensity along the propagation direction (corresponding to Fig 1 (c)). (g) Measured the axial FWHM of the overall PSF and propagation length for the different beams.

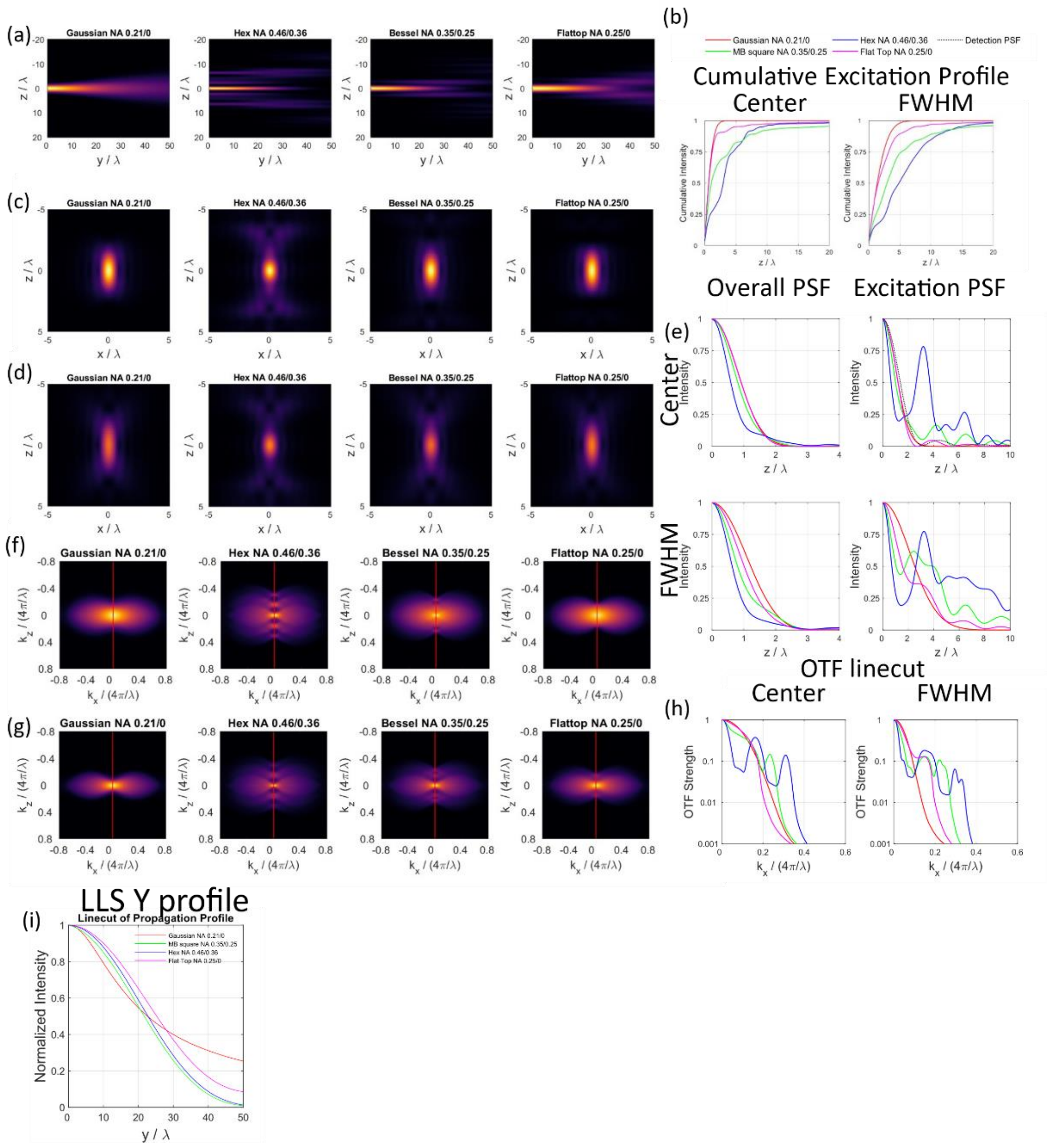

Figure SI3: Beam characterizations comparing Gaussian, MB square lattice, hexagonal lattice and flat top beams with the same propagation length of 20 µm. (a) Beam propagation profile (YZ profile) similar to Fig 1(b). (b) Cumulative excitation profile at the beam focus (left column) and at the propagation FWHM (right column). (c) Overall PSF for different beams at beam focus (similar to Fig 1(f)). (d) Overall PSF for different beams at the propagation FWHM (similar to Fig 1(g)). (e) Corresponding axial line cut of intensity profile in overall PSF (left column, as Fig 1 (h) and (i)) and in excitation profile (right column, as Fig 1 (d)) at the beam focus (top row) and FWHM of the propagation (bottom row). (f) Overall OTF for different beams at the beam focus (similar to Fig 2(a)). (g) Overall OTF for different beams at the propagation FWHM (similar to Fig 2(c)). (h) OTF line cut corresponding to Fig 2(e) and (f) for the different beams at the beam focus (left column) and FWHM of the propagation (right column). (i) Line cut at $z = 0$ along the propagation direction in (a) (similar to Fig 1(c)).

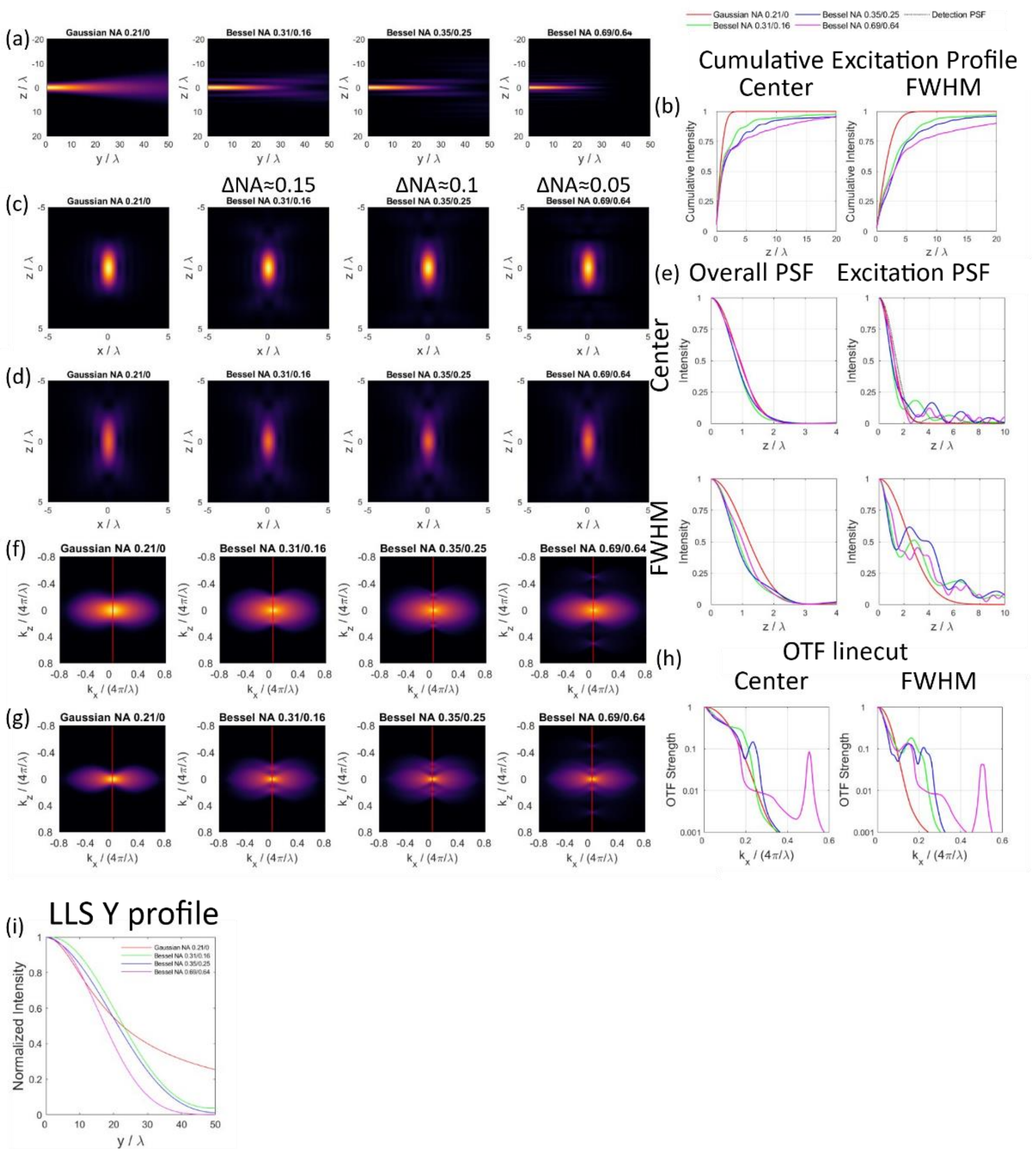

Figure SI4: Beam characterizations comparing Gaussian and different MB square beams with the same propagation length of 20 μm. (a) Beam propagation profile (YZ profile) similar to Fig 1(b). (b) Cumulative excitation profile at the beam focus (left column) and at the propagation FWHM (right column). (c) Overall PSF for different beams at beam focus (similar to Fig 1(f)). (d) Overall PSF for different beams at the propagation FWHM (similar to Fig 1(g)). (e) Corresponding axial line cut of intensity profile in overall PSF (left column, as Fig 1 (h) and (i)) and in excitation profile (right column, as Fig 1 (d)) at the beam focus (top row) and FWHM of the propagation (bottom row). (f) Overall OTF for different beams at the beam focus (similar to Fig 2(a)). (g) Overall OTF for different beams at the propagation FWHM (similar to Fig 2(c)). (h) OTF line cut corresponding to Fig 2(e) and (f) for the different beams at the beam focus (left column) and FWHM of the propagation (right column). (i) Line cut at *z* = 0 along the propagation direction in (a) (similar to Fig 1(c)).

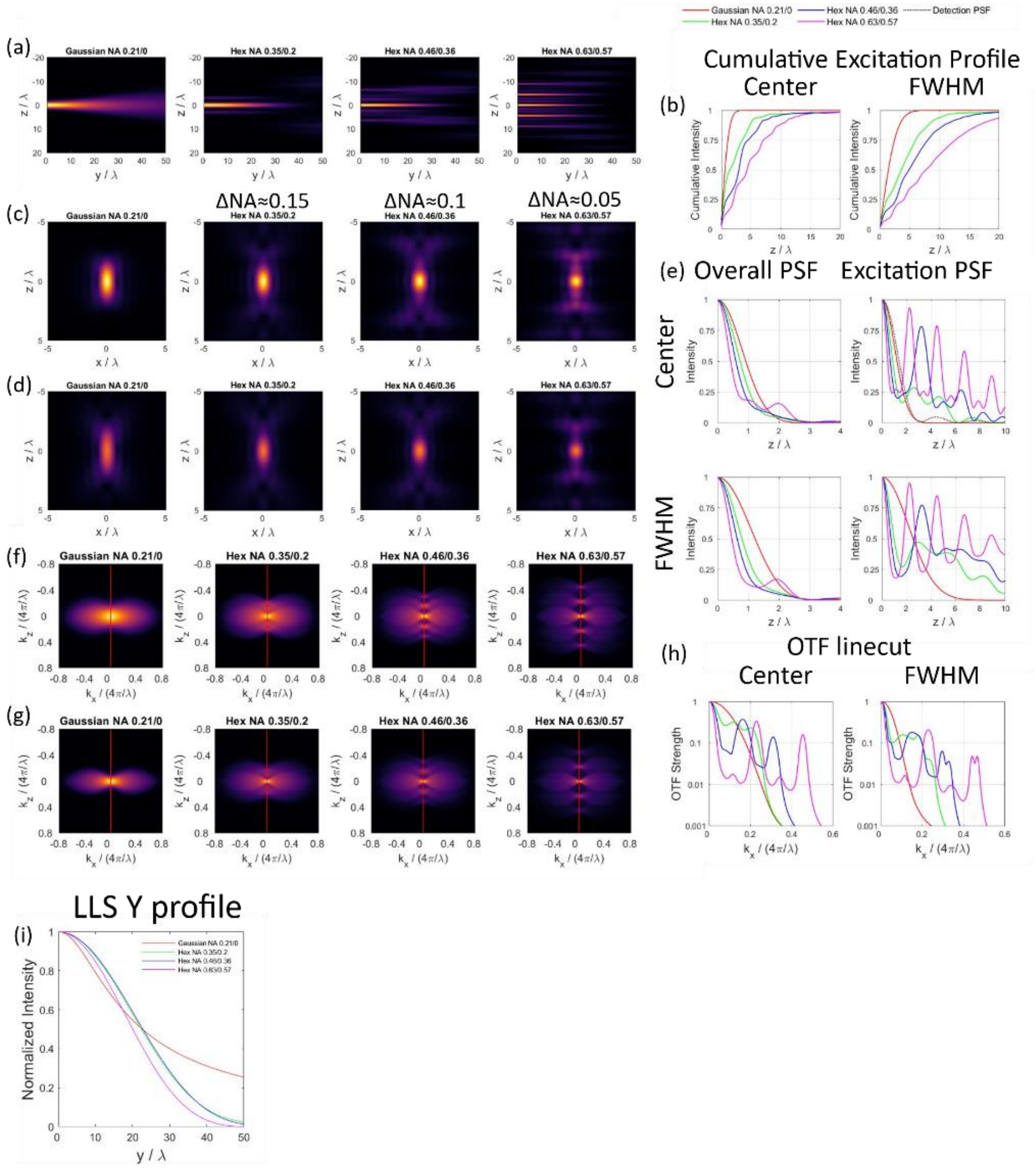

Figure SI5: Beam characterizations comparing Gaussian and different hexagonal lattice beams with the same propagation length of 20 µm. (a) Beam propagation profile (YZ profile) similar to Fig 1(b). (b) Cumulative excitation profile at the beam focus (left column) and at the propagation FWHM (right column). (c) Overall PSF for different beams at beam focus (similar to Fig 1(f)). (d) Overall PSF for different beams at the propagation FWHM (similar to Fig 1(g)). (e) Corresponding axial line cut of intensity profile in overall PSF (left column, as Fig 1 (h) and (i)) and in excitation profile (right column, as Fig 1 (d)) at the beam focus (top row) and FWHM of the propagation (bottom row). (f) Overall OTF for different beams at the beam focus (similar to Fig 2(a)). (g) Overall OTF for different beams at the propagation FWHM (similar to Fig 2(c)). (h) OTF line cut corresponding to Fig 2(e) and (f) for the different beams at the beam focus (left column) and FWHM of the propagation (right column). (i) Line cut at $z = 0$ along the propagation direction in (a) (similar to Fig 1(c)).

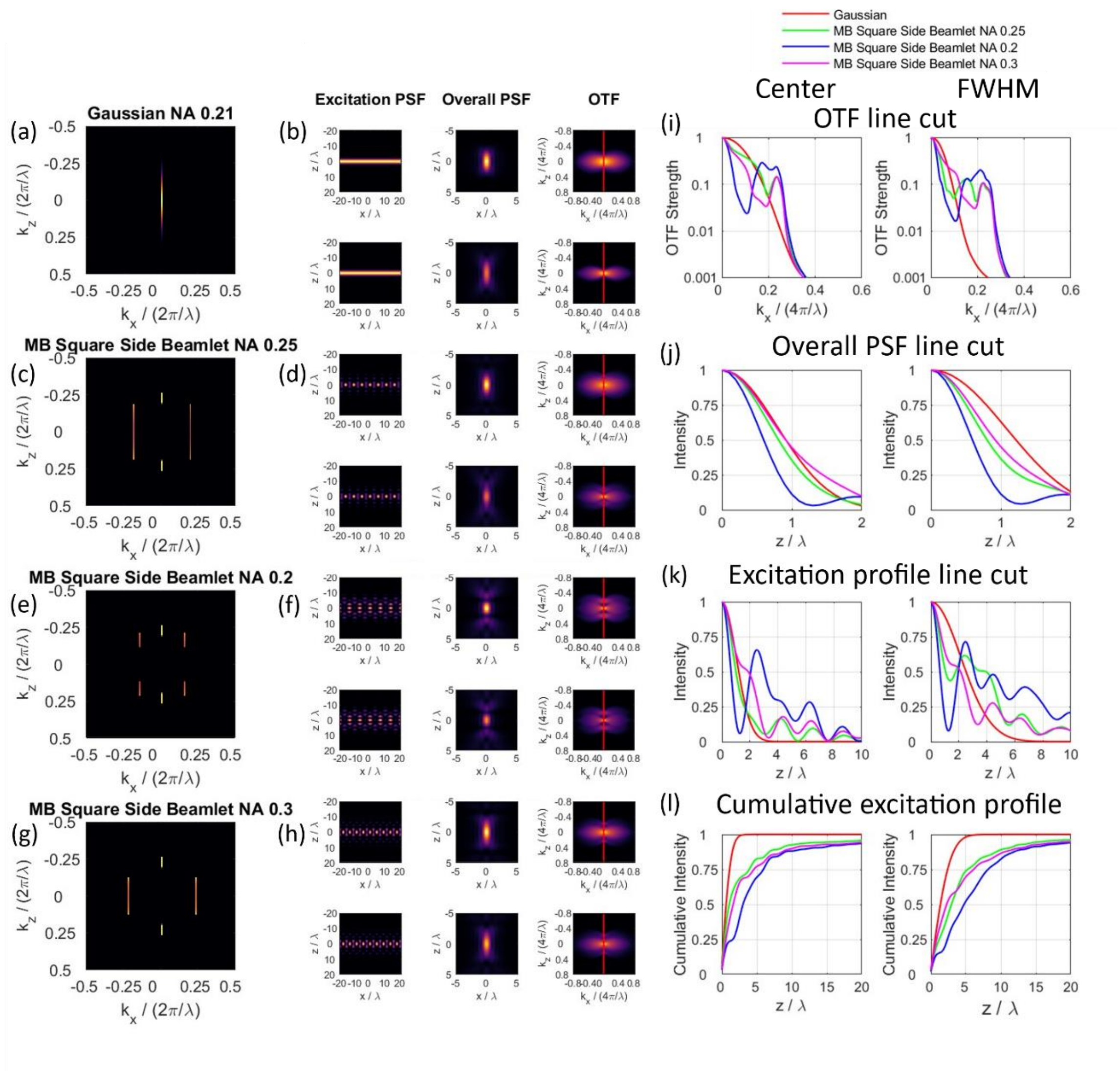

Figure SI6: Comparison of Gaussian with MB square lattice with the same NA but different spacing in the Bessel array. (a) pattern at the back pupil for the Gaussian beam. (b) Excitation profile (left column), overall PSF (middle) and overall OTF (right) at the center of the propagation (top row) and FWHM of the propagation (bottom row) for the Gaussian beam. (c) – (d) same as (a) and (b) for MB square lattice with the two side beamlets centered at NA = 0.25, which inscribes at the inner annulus. (e) – (f) same as (a) and (b) for MB square lattice with the two side beamlets centered at NA = 0.2, which get clipped by the inner annulus. (g) – (h) same as (a) and (b) for MB square lattice with the two side beamlets centered at NA = 0.3, which extends beyond the inner annulus. (i) – (l) characterizations of Gaussian and the different MB square beams in both real and frequency space at the propagation center (left column) and the propagation FWHM (right column). (i) OTF axial line-cut along k_x=0 for the different beams (corresponding to Fig 2(e) and (f)). (j) Overall PSF axial line cut along x = 0 for the different beams (corresponding to Fig 1(h) and (i)). (k) excitation profile axial line-cut along x = 0 for the different beams (corresponding to Fig 1(d)). (l) cumulative intensity profile along x = 0 for the excitation profile (corresponding to Fig 1(e)).

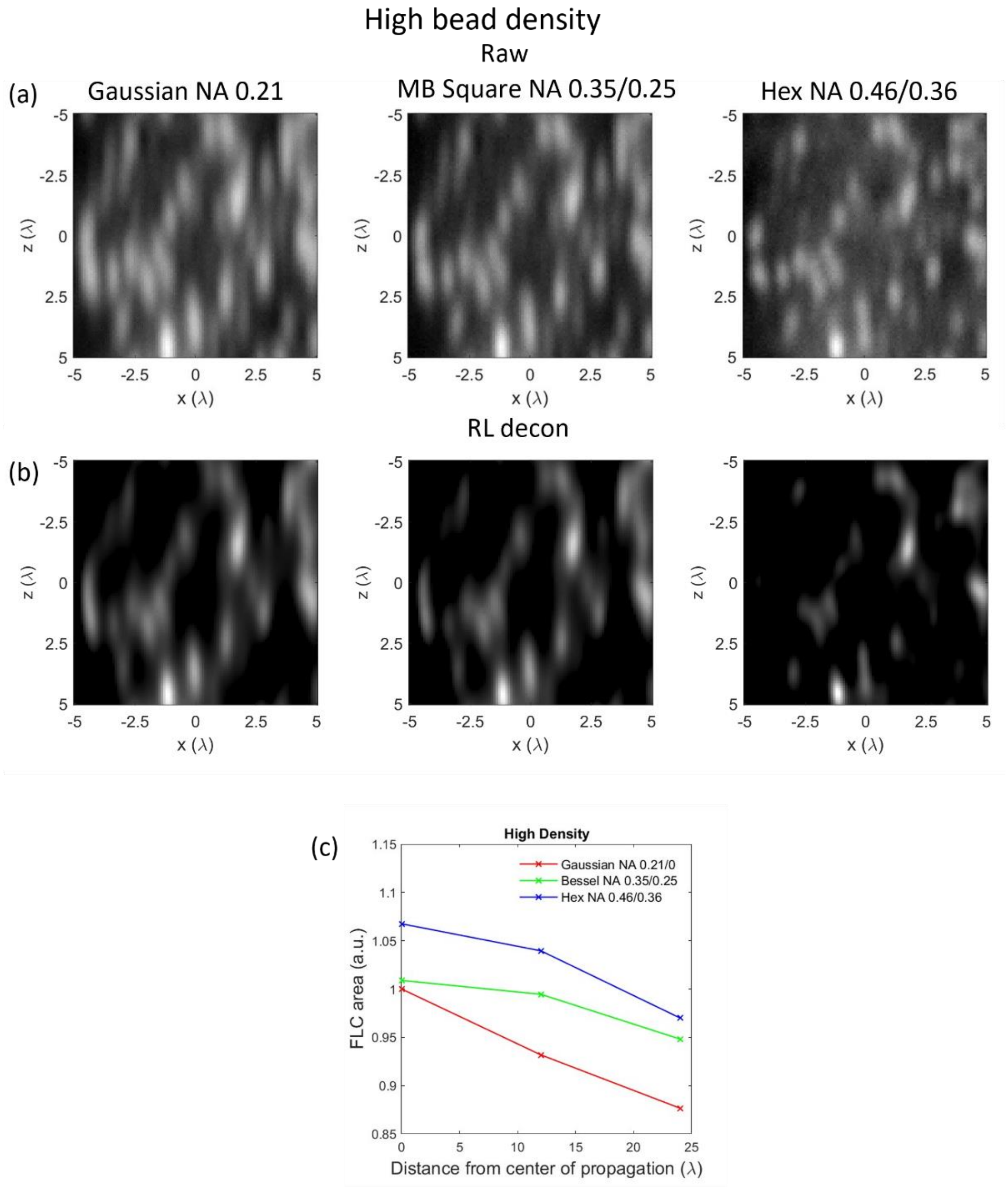

Figure SI7: Multi-emitter simulation for the three beam patterns in Fig 1 at the propagation center and FWHM with higher emitter density (10/um^3). (a) Raw simulated images for the different beams at the propagation center (corresponding to Fig 3 (a)). (b) Richardson-Lucy deconvolved images from (a). (c) Relative FPC area for the raw simulated images, corresponding to Fig 3 (e).

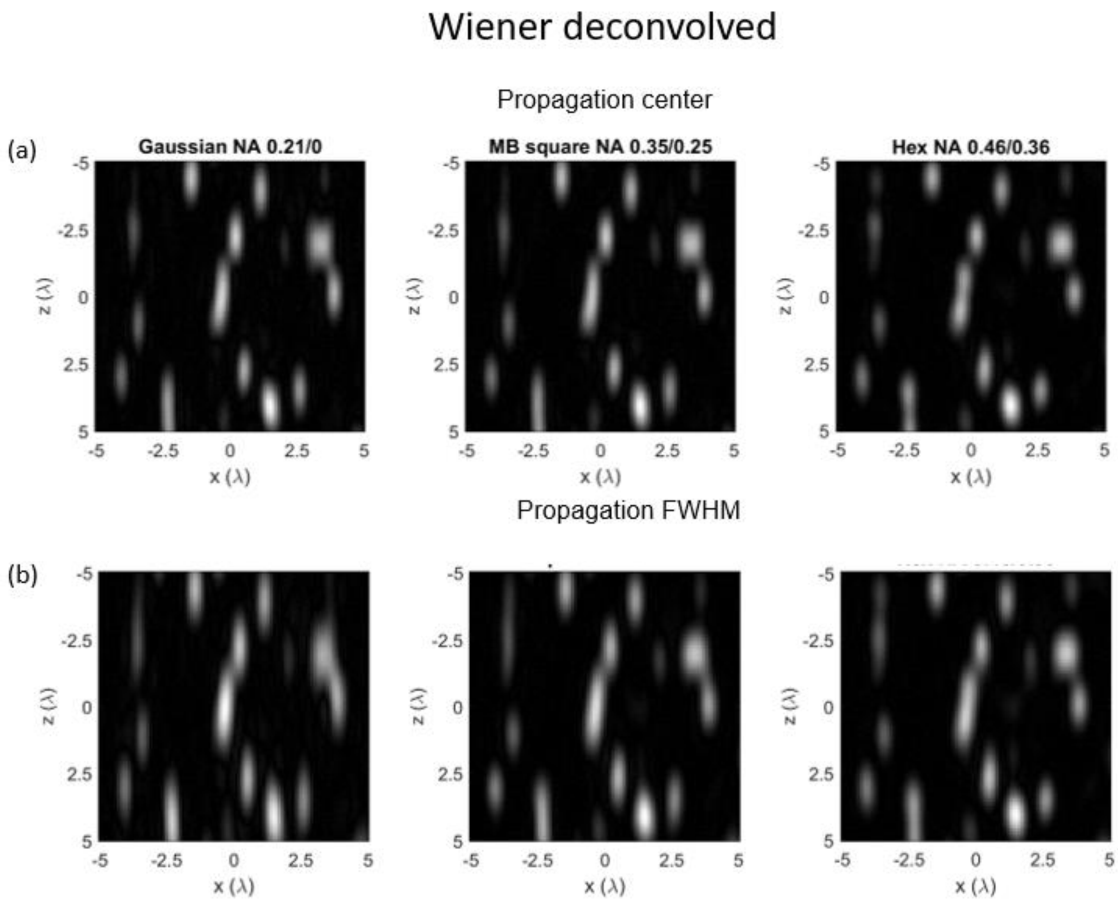

Figure SI8: Weiner deconvolved images for the raw multi-emitter simulation for different beam patterns at the propagation center (a) and FWHM (b) from Figure 3 (a) and (b)

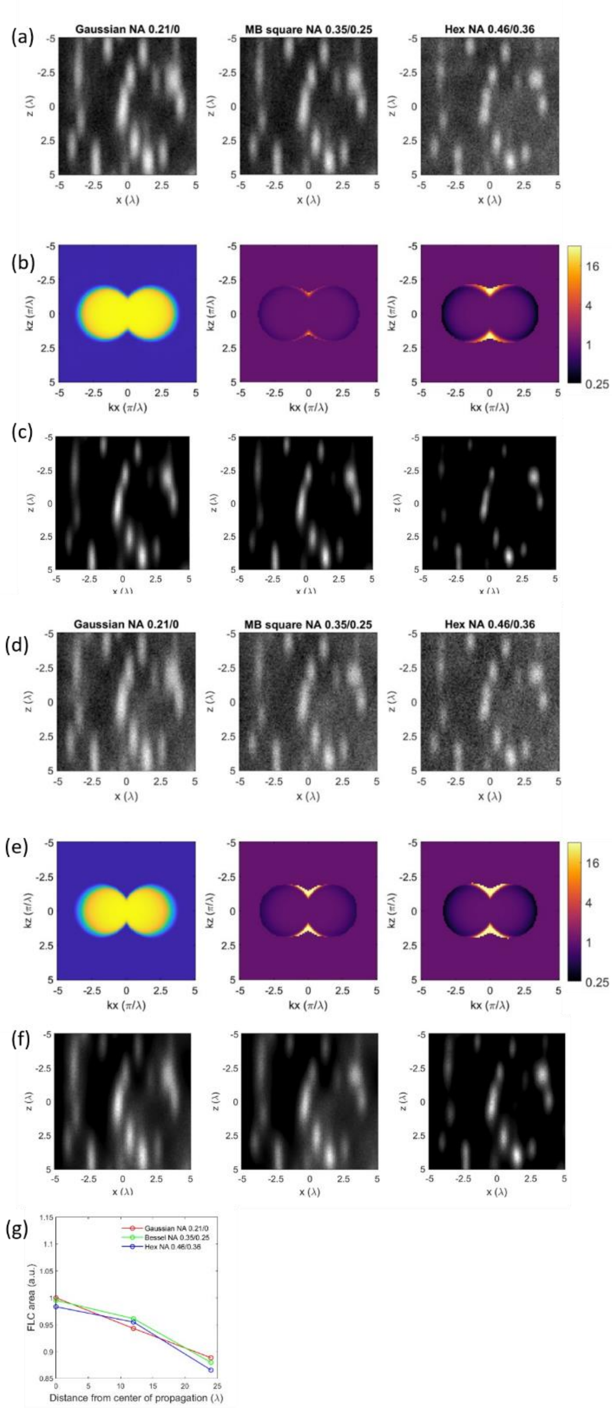

Figure SI9: Multi-emitter simulation for the three beam patterns in Fig 1 at the propagation center and FWHM with lower signal (~100 counts at peak). (a) Raw simulated images for the different beams at the propagation center (corresponding to Fig 3 (a)). (b) FPC based on raw images shown in (a), for MB-square lattice and hexagonal lattice beams the ratio relative to the Gaussian beam is shown (corresponding to Fig 3 (c)). (c) Richardson-Lucy deconvolved images from (a). (d) – (f) corresponding images for (a) - (c) for beams at the FWHM of the propagation. (g) Relative FPC area for the raw simulated images, corresponding to Fig 3 (e).

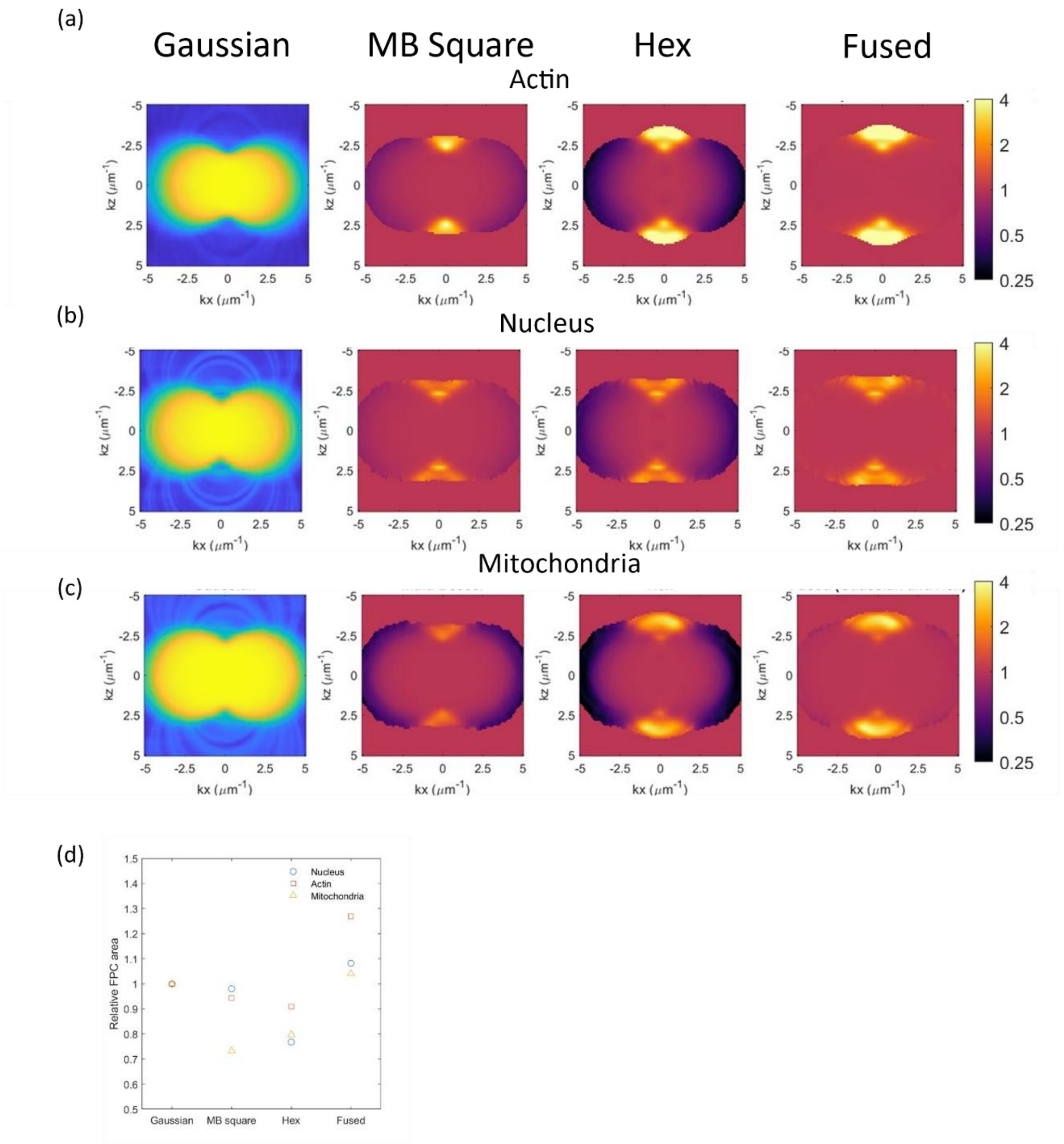

Figure SI10: Quantitative comparison between Gaussian, MB square lattice, hexagonal lattice and the fused condition based on Fourier plane correlation (FPC) graph. (a) FPC graph based on raw images for actin of different beams and a fused MB-square + hexagonal condition shown in Fig 4. For MB square, hexagonal lattice beams and the fused condition, the ratio between the corresponding Gaussian beam is used. (b) FPC graph for nucleus of different beams. (c) FPC graph for mitochondria of different beams. (d) Relative FPC area for the three different beams and the fused condition in actin, nucleus and mitochondria.

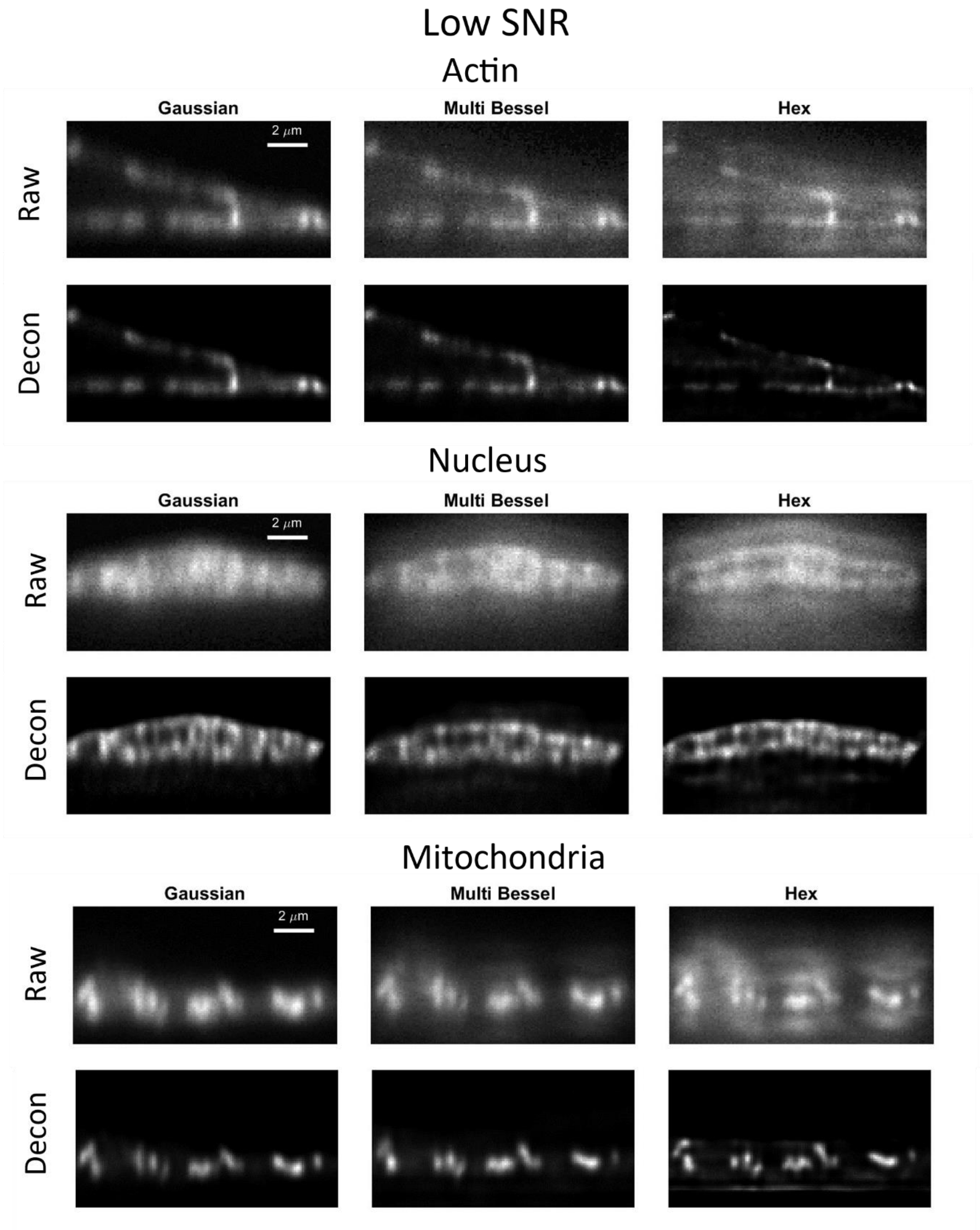

Figure SI11 Raw and deconvolved YZ slices of different beams with lower signal-to-noise ratio on actin, nucleus and mitochondria.

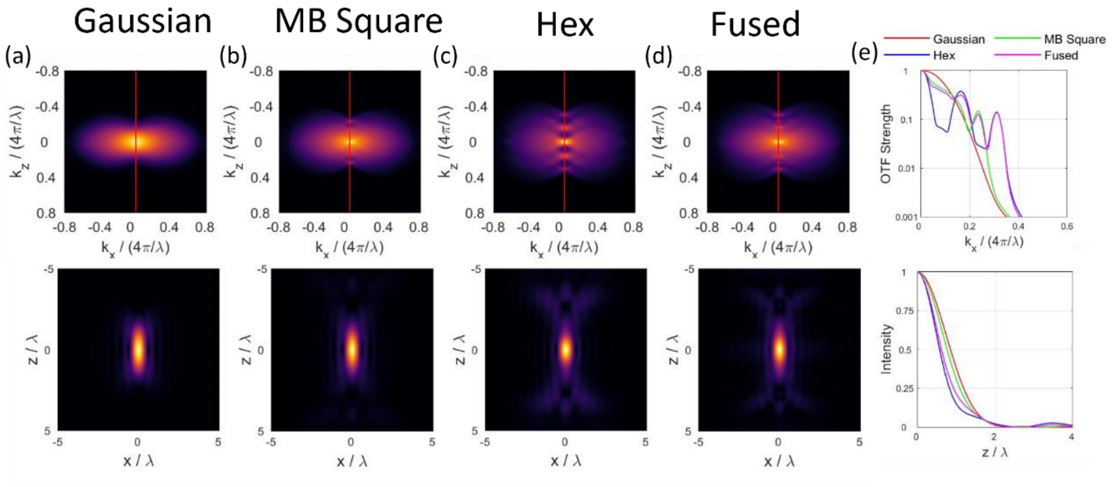

Figure SI12 Simulation comparison between Gaussian, MB square lattice, hexagonal lattice beam and the fused condition (a) OTF (top) and PSF (bottom) at the propagation center for Gaussian beam. (b) OTF (top) and PSF (bottom) at the propagation center for MB square lattice beam. (c) OTF (top) and PSF (bottom) at the propagation center for hex beam. (d) OTF (top) and PSF (bottom) at the propagation center for fusion between MB square lattice and hex lattice. (e) OTF line cut at $k_x = 0$ (top) and PSF line cut at $x = 0$ (bottom) comparing Gaussian, MB square, hex and fused beams.

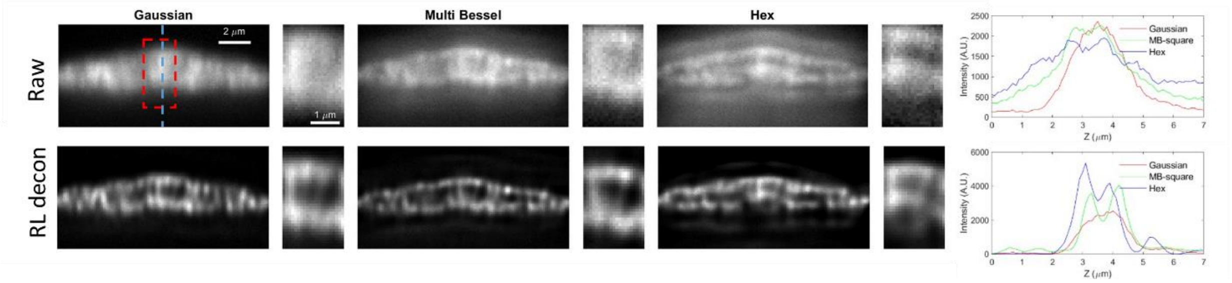

Figure SI13 Raw and deconvolved YZ slices of different beams on nucleus. Hex beams show visible artifacts due to sample-induced aberrations. Graph organization follows the same as Fig 4.

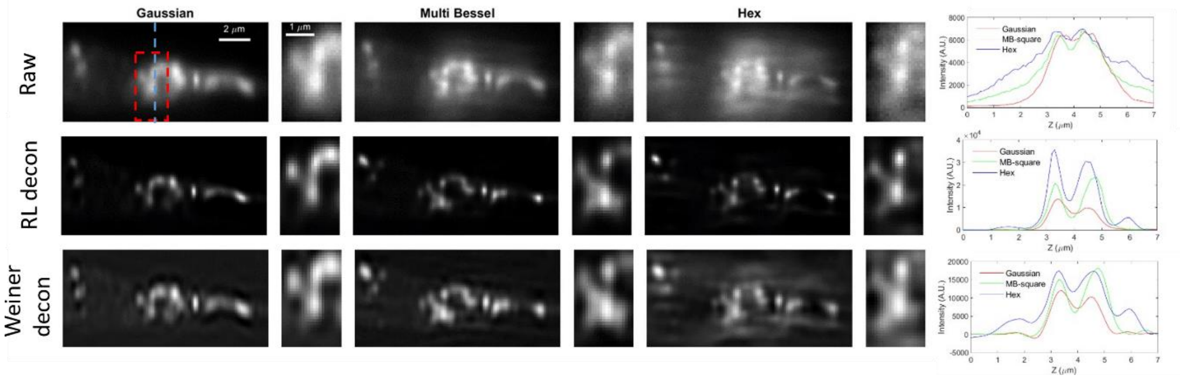

Figure SI14 Comparison between raw, Richard-Lucy deconvolved and Wiener deconvolved images for the three different beams of mitochondria. Deconvolution methods are as described in the method section. Graph organization follows the same as Fig 4.

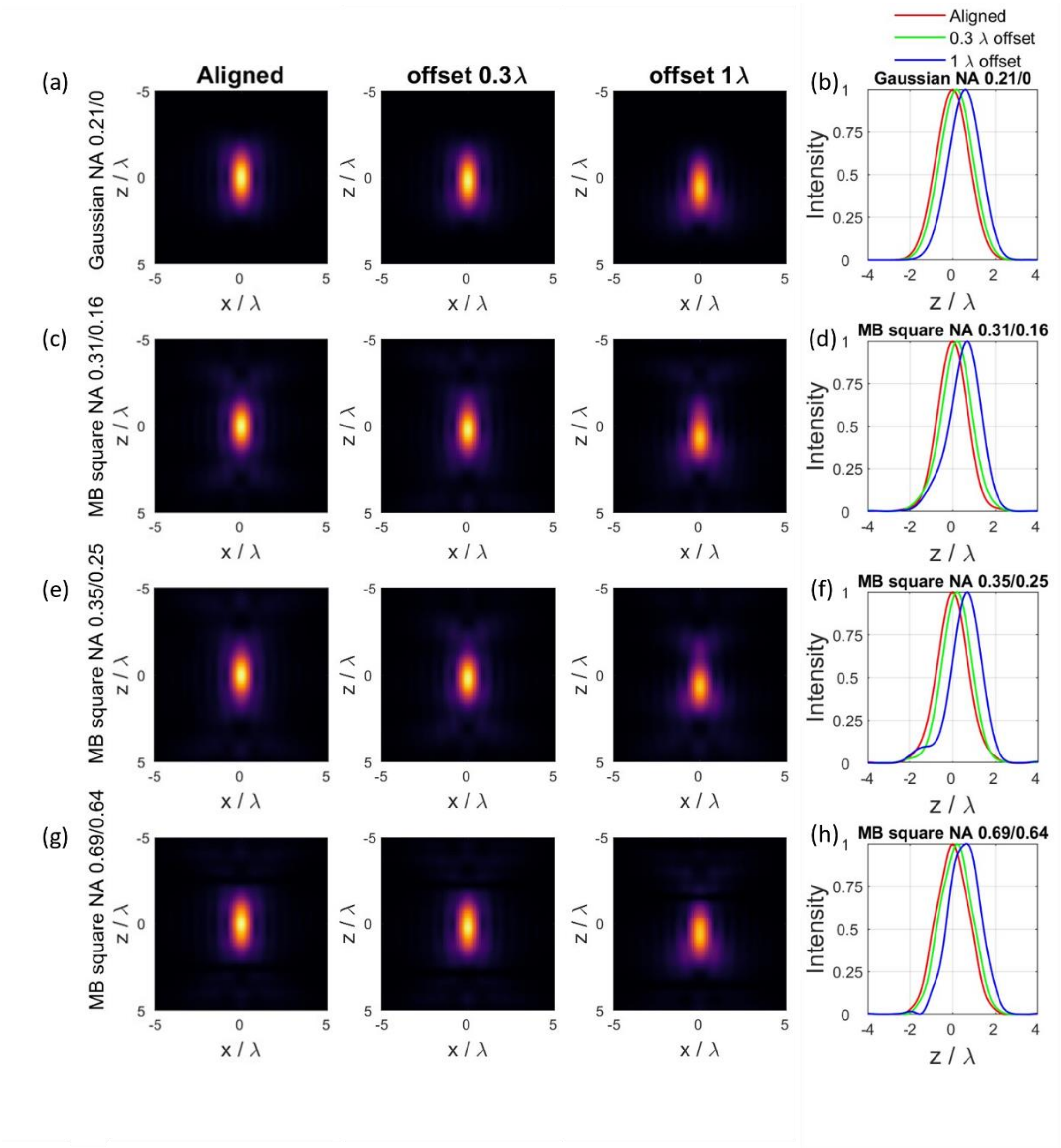

Figure SI15 Aberration from excitation focus offset on MB square lattice beam. (a) Overall PSF for the Gaussian beam with either 0 (left column), 0.3 λ (middle column) and 1 λ (right column) offset from detection focal plane (z axis). (b) Overall PSF line cut at $x = 0$. (c) – (d) Overall PSF and PSF line cut similar to (a) and (b) for MB square lattice beam with NA of 0.31/0.16 (ΔNA ≈ 0.15). (e) – (f) Overall PSF and PSF line cut similar to (a) and (b) for MB square lattice beam with NA of 0.35/0.25 (ΔNA ≈ 0.1). (g) – (h) Overall PSF and PSF line cut similar to (a) and (b) for MB square lattice beam with NA of 0.69/0.64 (ΔNA ≈ 0.05).

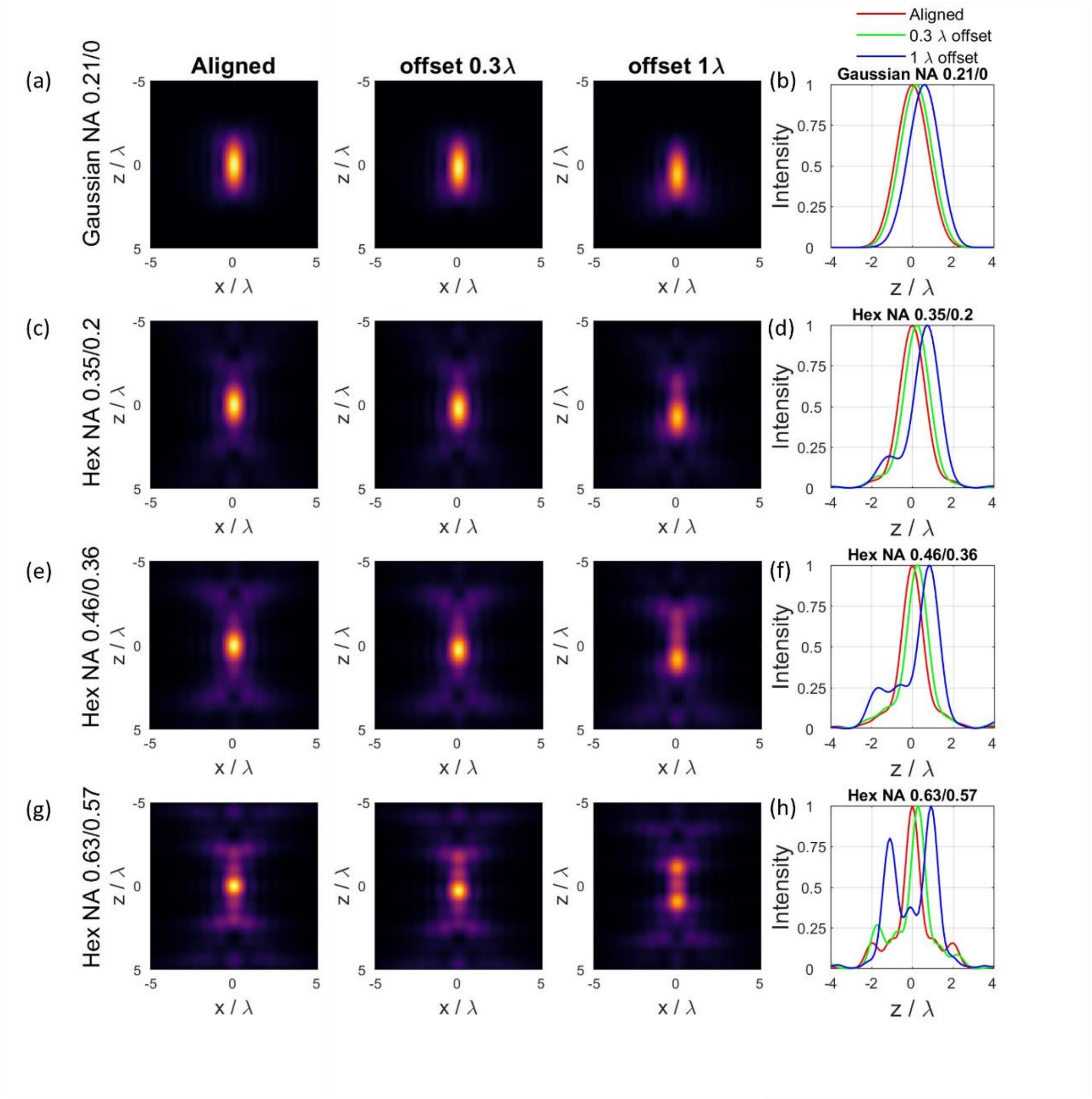

Figure SI16 Aberration from excitation focus offset on hexagonal lattice beam. (a) Overall PSF for the Gaussian beam with either 0 (left column), 0.3 λ (middle column) and 1 λ (right column) offset from detection focal plane (z axis). (b) Overall PSF line cut at $x = 0$. (c) – (d) Overall PSF and PSF line cut similar to (a) and (b) for Hex beam with NA of 0.35/0.2 (ΔNA ≈ 0.15). (e) – (f) Overall PSF and PSF line cut similar to (a) and (b) for Hex beam with NA of 0.46/0.36 (ΔNA ≈ 0.1). (g) – (h) Overall PSF and PSF line cut similar to (a) and (b) for Hex with NA of 0.63/0.57 (ΔNA ≈ 0.05).

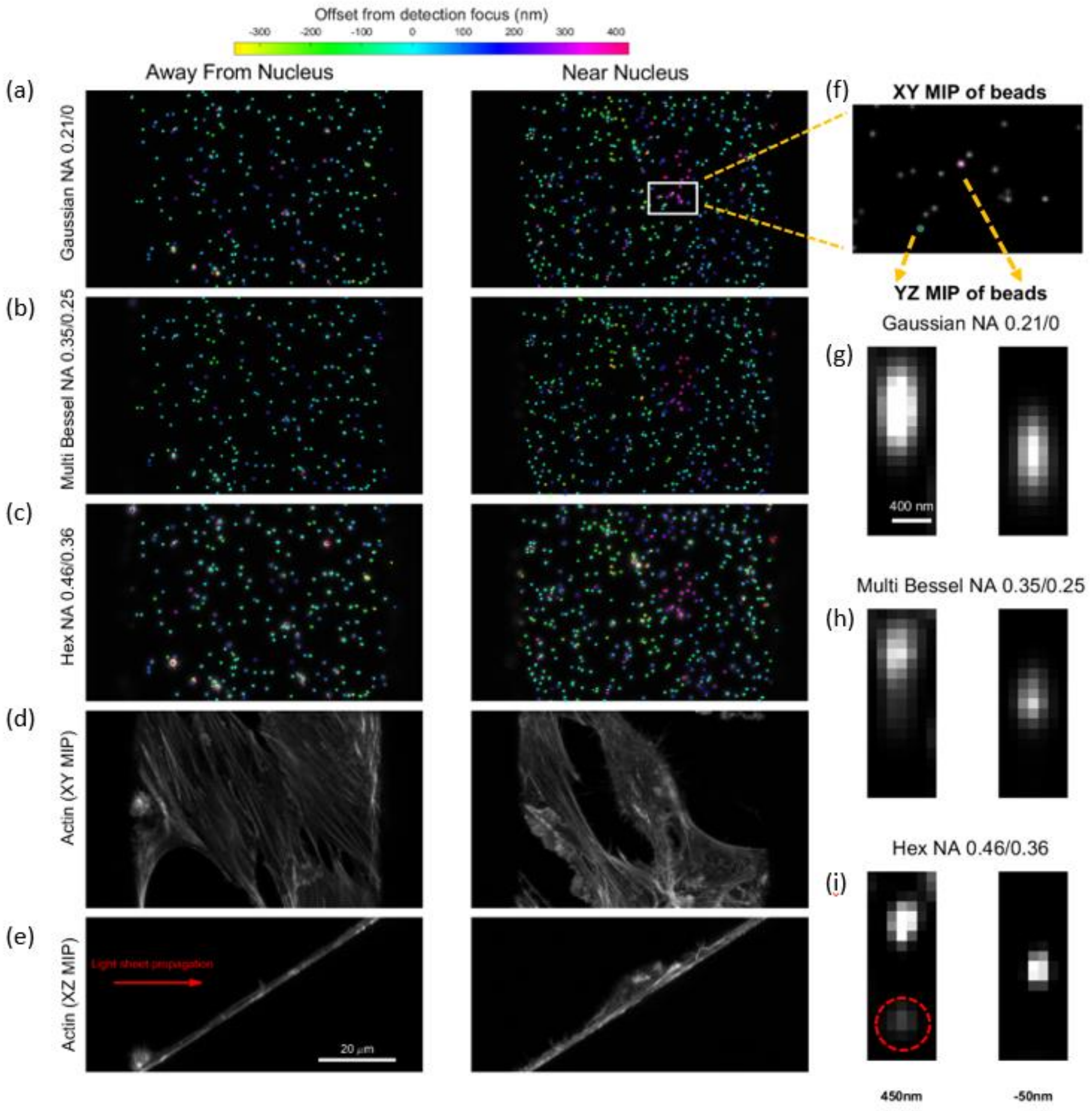

Figure SI17 Experimental measurements of sample induced light sheet offset. (a) Measurements of Gaussian light sheet offset from detection focus based on beads underneath COS7 cells. The left column shows a region far away from the cell nucleus, and the right column shows a region close to the cell nucleus. (b) Measurements of MB square lattice light sheet offset from detection focus. (c) Measurements of hexagonal light sheet offset from detection focus. (d) Lateral maximum intensity projection (XY MIP) of actin. (e) XZ MIP of actin. The red arrow indicates the propagation direction of the light sheet. (f) Zoomed XY MIP for the region within the white box in (a). (g)-(i) Zoomed YZ MIP of two sample bead where the light sheet is not deflected (left column, corresponds to the green circle in (f)) and deflected (right column, corresponds to the purple circle in (f)) taken with Gaussian beam (g), MB square lattice beam (h) and hex beam (i). The ghosting artifact caused by the light sheet offset in hexagonal beam condition is highlighted with the dashed red circle in (i).

(a)

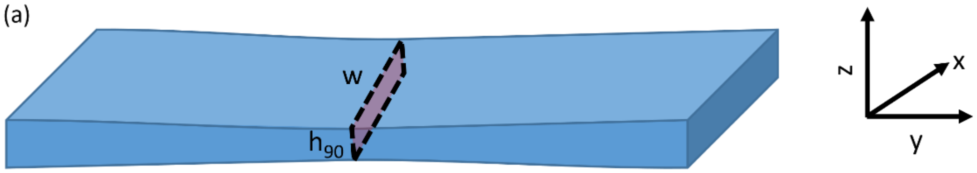

(b)

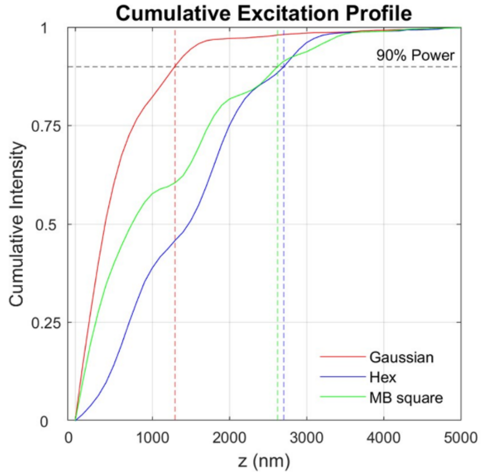

Figure SI18 Estimation of the average power of the light sheet at the sample. (a) Sketch of illumination profile of light sheet. The average power is estimated by the total light sheet power (determined by power measured at the back pupil of the excitation objective and the transmission efficiency of the objective) and the cross-section area (black dashed box). The area is the product of the width of the light sheet (w, which is ~ 200µm for all three light sheet patterns) and the height within which 90% of the total power is enclosed (h_{90}). (b) Cumulative excitation profile for the Gaussian, MB square lattice and hexagonal lattice (similar as Fig 1 (e)). The horizontal dashed line indicates 90% of the cumulative power, and the vertical dashed line indicates the width within which 90% of the power is enclosed for each beam (h_{90}).

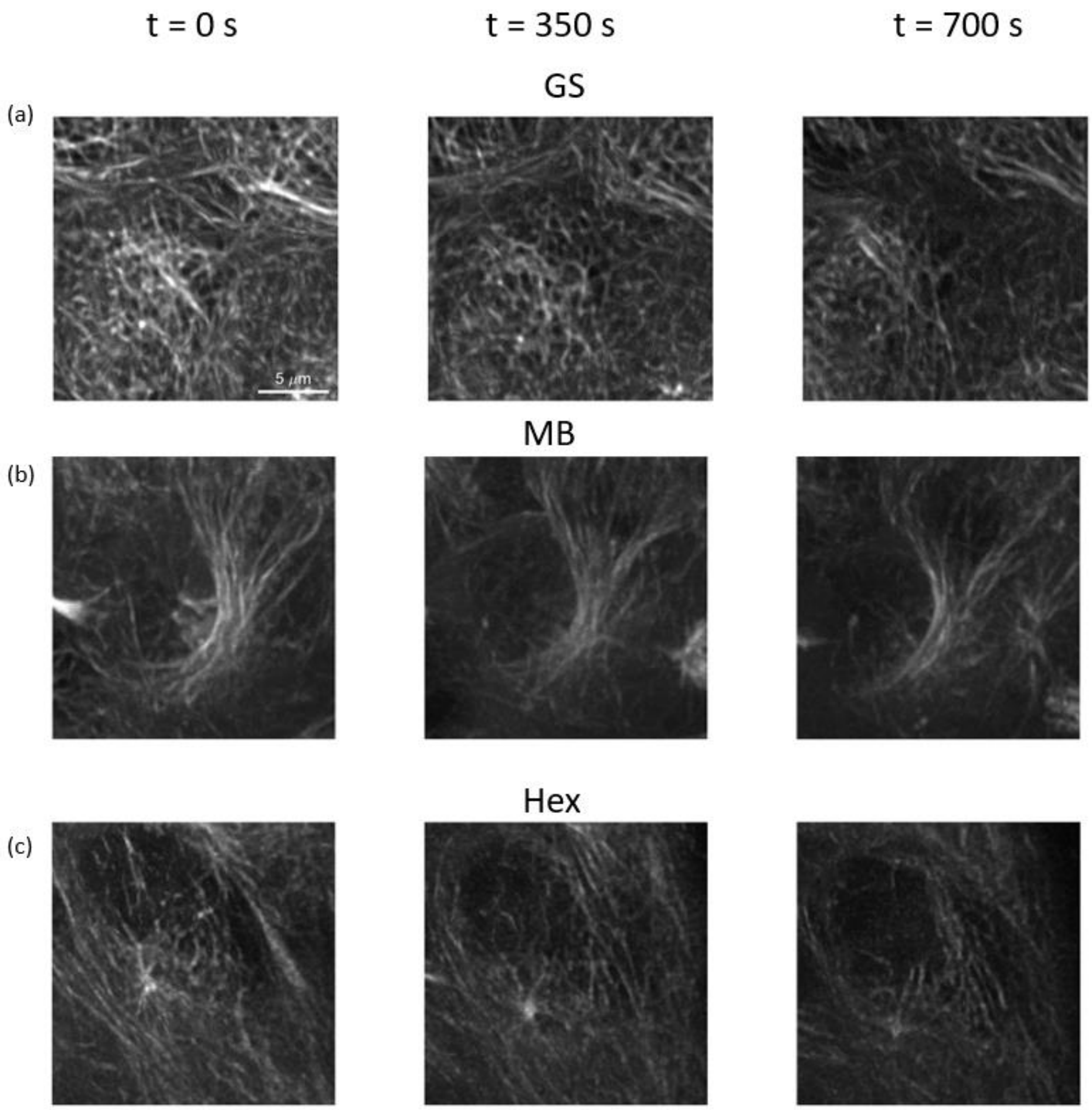

Figure SI19 Time lapse for microtubules in live IPSC cells. For a given lightsheet, the same field of view is imaged for 700 s at a rate of 0.14 Hz, with sample XY maximum intensity projection at t = 0 s (left column), t = 350 s (middle column) and t = 700 s (right column). The same intensity scaling is applied to all three images within the same time lapse. (a) Gaussian beam. (b) MB square lattice beam. (c) Hexagonal lattice beam.

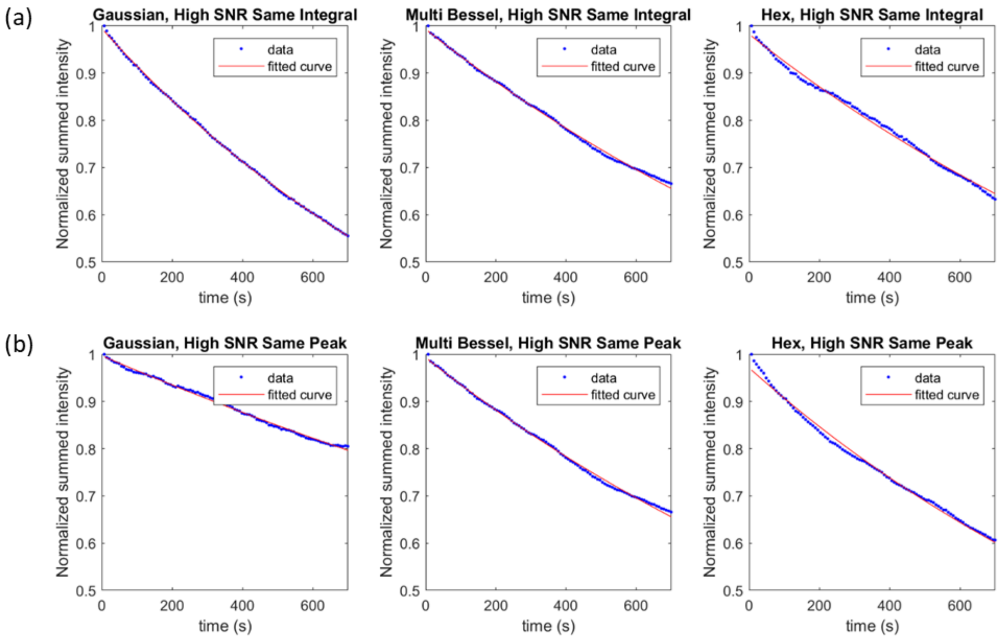

Figure SI20 Comparison of photo bleaching curves for different light sheets. (a) Relative summed intensity against time for Gaussian, MB square lattice and hexagonal lattice beams with the same integrated intensity based on bead calibration (see methods for details). The relative summed intensity is calculated based on the pixel wise summed intensity after subtracting the dark current and then normalized by the summed intensity of the first frame (blue dots). The photo bleaching curves are fitted by an exponential decay (red line). (b) Relative summed intensity against time for the same three beams with the same peak intensity.

(a)

(b)

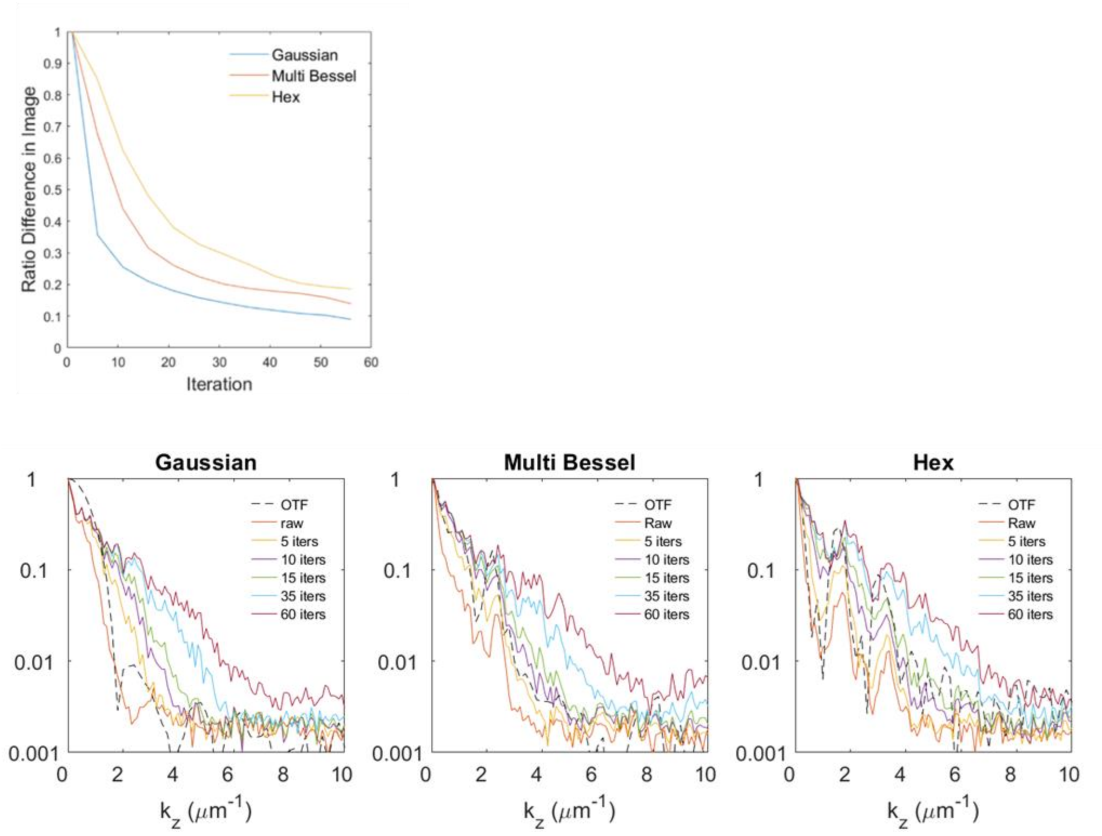

Figure SI21: Dependence on the number of Richard-Lucy deconvolution iterations for Gaussian, MB square lattice and hexagonal lattice. (a) Convergence rates of Richard-Lucy deconvolution for different beams. The pixel-wise root mean square differences relative to the first iteration of Richard-Lucy deconvolution is plotted against iterations for the three different beams. (b) Comparison of the theoretical OTF support, the OTF of the raw images, and Richard-Lucy deconvolved images with different iterations of different beams of actin.

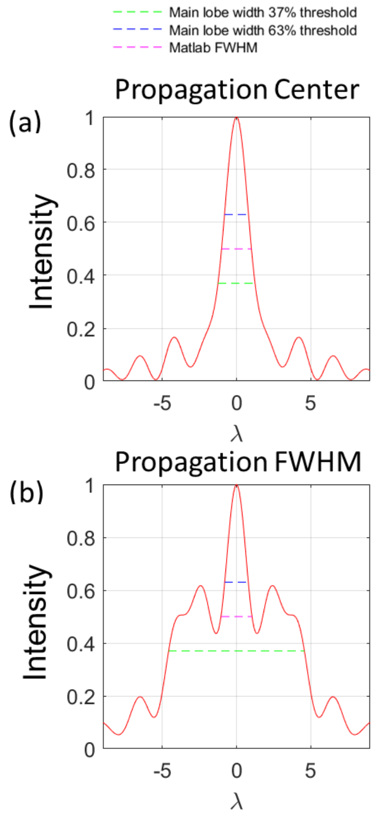

(c)

	**Propagation Center (λ)**	**Propagation FWHM (λ)**
Main lobe with 37% threshold	2.50	9.15
Main lobe with 63% threshold	1.62	1.59
Matlab FWHM	2.01	2.10

Figure SI22: Comparison of different metrics for characterizing the width of the excitation profile peak in real space. (a) Excitation profile at the beam focus. The green and blue dashed lines indicate the width where the intensity drops to 37% (same as the main lobe width defined in Remacha et al) and 63% of the peak respectively. The magenta dashed line indicates the full width half max (FWHM) calculated by the "findpeaks" function in Matlab (Mathworks). (b) Similar to (a) but measured at the FWHM of beam propagation (10 µm from center). (c) Table for the width of the peak characterized by different metrics.

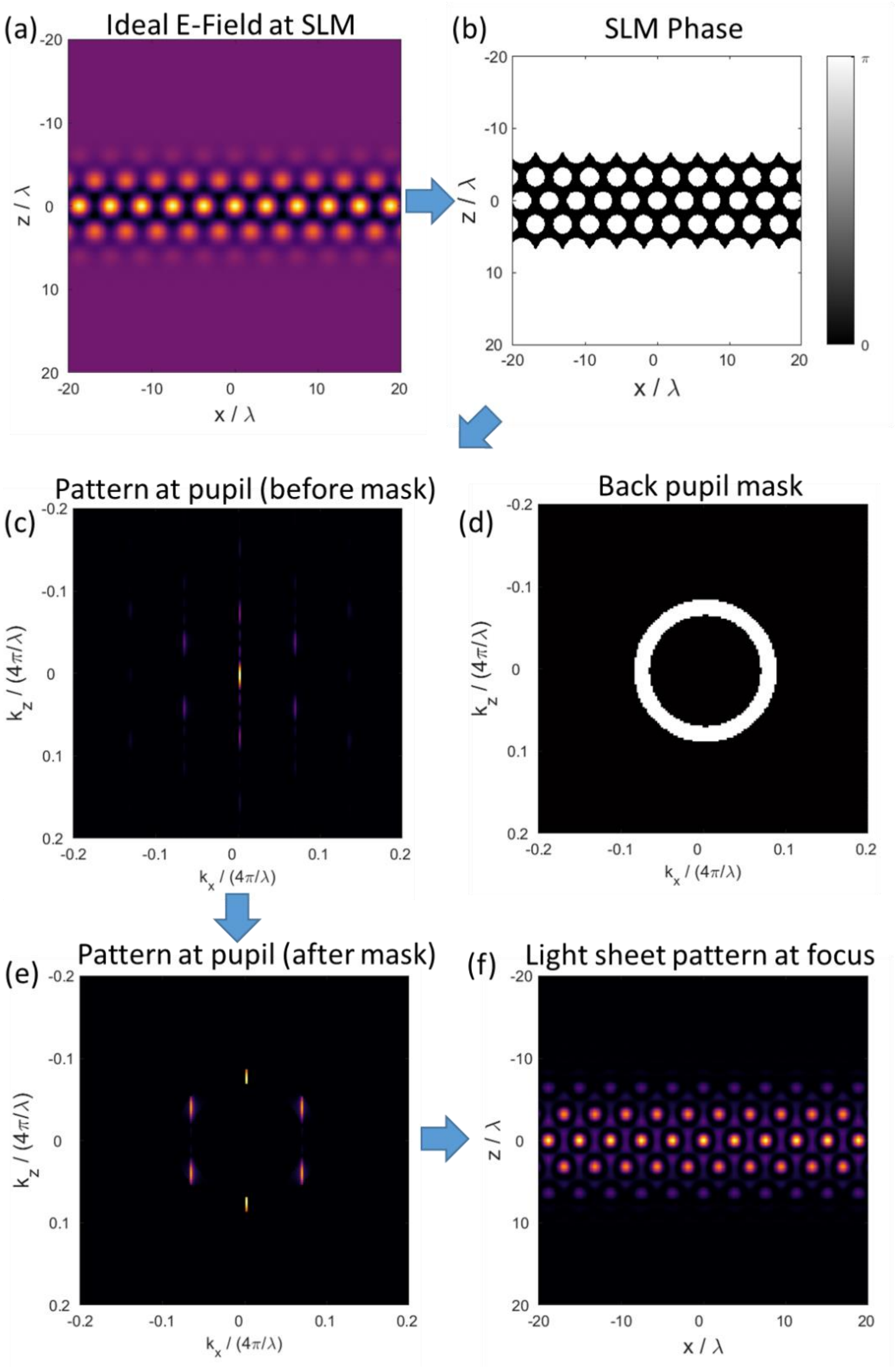

Figure SI23: Simulation process for hexagonal lattice beam. (a) Simulated ideal electric field at the sample and SLM for a Hex beam with NA 0.46/0.36. (b) Binary phase of the electric field at the sample and SLM. (c) Simulated illumination pattern prior to annular mask, which is the Fourier transform of the pattern at the SLM in (b). (d) Simulated annular mask, with the same NA_{min} and NA_{max} as the simulated pattern at the back pupil. (e) Simulated illumination pattern after filtering by the annular mask. (f) Final simulated intensity pattern at the sample plane. This is squared Fourier transform of the pattern in (e).

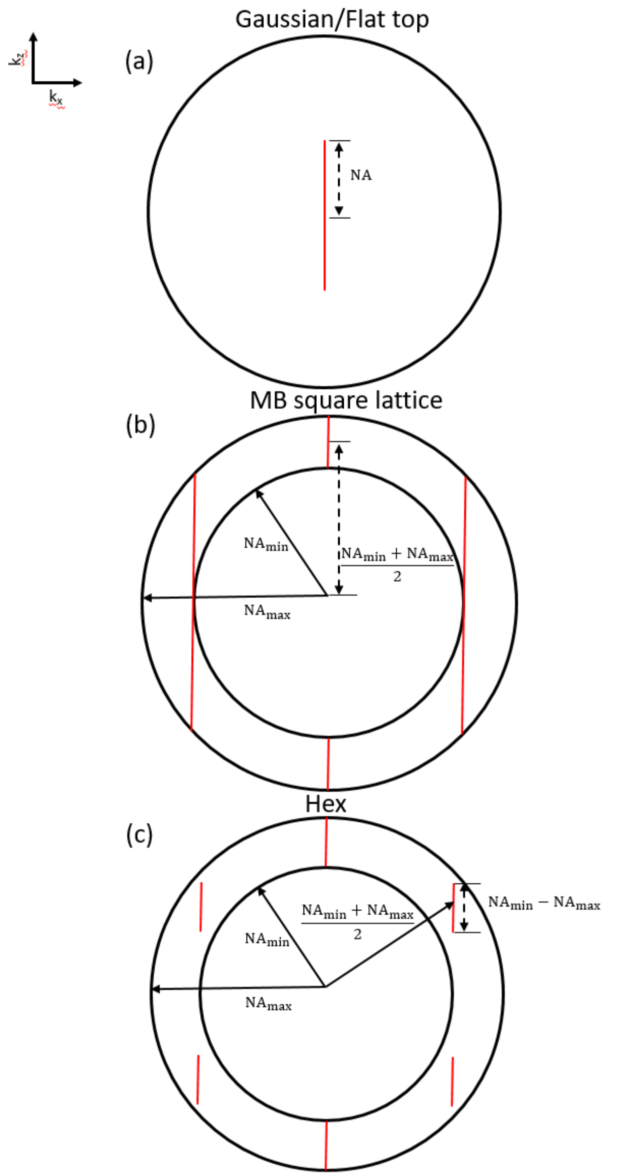

Figure SI24: Schematic drawing of back pupil for simulated Gaussian, MB square lattice and hexagonal beams. (a) Schematic drawing of back pupil for Gaussian and flat top beams. For flat top, the intensity at the back pupil follows a square function with cut-off at k_z = NA (k_z is already normalized as $\frac{1}{\text{length}}/\frac{1}{\lambda}$, and is thus dimensionless). For Gaussian beam, the E field profile at the back pupil follows $E = E_0 * \exp(-(\frac{k_z}{NA})^2)$. (b) Schematic drawing of back pupil for MB-square lattice. NA_{min} and NA_{max} indicates the minimum and maximum NA. The two beamlets at k_x = 0 (red lines at 0 and 12 o'clock direction) are centered at NA = $\frac{NA_{min}+NA_{max}}{2}$, and the two beamlets at k_z = 0 (red lines at 3 and 9 o'clock direction) are centered at NA = NA_{min}. All beamlets are extended along k_z until they are clipped by NA_{min} or NA_{max}. (c) Schematic drawing of back pupil for Hex lattice. Notation are the same as in (a). All beamlets are uniformly distributed along the back pupil, and locate at NA = $\frac{NA_{min}+NA_{max}}{2}$. The extension of all beamlets along k_z are $NA_{max} - NA_{min}$.
