# Supplementary material for: A quantitative analysis of various patterns applied in lattice light sheet microscopy
Source: Nat Commun. 2022 Aug 8;13:4607. doi: 10.1038/s41467-022-32341-w (PMC9360440; doi:10.1038/s41467-022-32341-w)
Supplement: Supplementary file 2 — Reporting Summary [file 41467_2022_32341_MOESM2_ESM.pdf]

nature portfolio

Corresponding author(s): Wesley R. Legant

Last updated by author(s): Jul 22, 2022

Reporting Summary

Nature Portfolio wishes to improve the reproducibility of the work that we publish. This form provides structure for consistency and transparency in reporting. For further information on Nature Portfolio policies, see our Editorial Policies and the Editorial Policy Checklist.

Statistics

For all statistical analyses, confirm that the following items are present in the figure legend, table legend, main text, or Methods section.

n/a	Confirmed	
☐	☒	The exact sample size (*n*) for each experimental group/condition, given as a discrete number and unit of measurement
☐	☒	A statement on whether measurements were taken from distinct samples or whether the same sample was measured repeatedly
☒	☐	The statistical test(s) used AND whether they are one- or two-sided<br>*Only common tests should be described solely by name; describe more complex techniques in the Methods section.*
☒	☐	A description of all covariates tested
☒	☐	A description of any assumptions or corrections, such as tests of normality and adjustment for multiple comparisons
☐	☒	A full description of the statistical parameters including central tendency (e.g. means) or other basic estimates (e.g. regression coefficient) AND variation (e.g. standard deviation) or associated estimates of uncertainty (e.g. confidence intervals)
☒	☐	For null hypothesis testing, the test statistic (e.g. *F*, *t*, *r*) with confidence intervals, effect sizes, degrees of freedom and *P* value noted<br>*Give P values as exact values whenever suitable.*
☒	☐	For Bayesian analysis, information on the choice of priors and Markov chain Monte Carlo settings
☒	☐	For hierarchical and complex designs, identification of the appropriate level for tests and full reporting of outcomes
☒	☐	Estimates of effect sizes (e.g. Cohen's *d*, Pearson's *r*), indicating how they were calculated

Our web collection on statistics for biologists contains articles on many of the points above.

Software and code

Policy information about availability of computer code

Data collection	Experimental images were acquired on a custom built lattice light sheet microscope as described in Methods. For simulation and image analysis, we used Matlab (version 2021b) to generate light sheet point spread functions and simulated images.
Data analysis	For point spread function characterization, deconvolution, Fourier plane correlation, and photobleaching rate calculation, we used customized code in Matlab (version 2021b). The source code generated during and/or analyzed during the current study are available at: https://github.com/legantlab/Shi_et_al_Nat_Comm_SourceCode. Code is provided under The MIT License for open source software.

For manuscripts utilizing custom algorithms or software that are central to the research but not yet described in published literature, software must be made available to editors and reviewers. We strongly encourage code deposition in a community repository (e.g. GitHub). See the Nature Portfolio guidelines for submitting code & software for further information.

Data

Policy information about availability of data

All manuscripts must include a data availability statement. This statement should provide the following information, where applicable:

- Accession codes, unique identifiers, or web links for publicly available datasets
- A description of any restrictions on data availability
- For clinical datasets or third party data, please ensure that the statement adheres to our policy

The datasets generated during and/or analyzed during the current study can either be regenerated from the available source code or are available from the corresponding author on request.

Human research participants

Policy information about studies involving human research participants and Sex and Gender in Research.

Reporting on sex and gender	Not applicable
Population characteristics	Not applicable
Recruitment	Not applicable
Ethics oversight	Not applicable

Note that full information on the approval of the study protocol must also be provided in the manuscript.

Field-specific reporting

Please select the one below that is the best fit for your research. If you are not sure, read the appropriate sections before making your selection.

- [x] Life sciences
- [ ] Behavioural & social sciences
- [ ] Ecological, evolutionary & environmental sciences

For a reference copy of the document with all sections, see nature.com/documents/nr-reporting-summary-flat.pdf

Life sciences study design

All studies must disclose on these points even when the disclosure is negative.

Sample size	No statistical method was used to predetermine the sample size.<br>5 different cells were taken for IPSC cells phototoxicity and photobleaching test in Figure 5. This sample size was sufficient to clearly demonstrate the trends in the data.<br>6 different cells (three each from two experiments) were taken for the fixed cell datasets in Figure 4. The size was sufficient to illustrate that the same trade-offs in image qualities are observed across different imaging modalities. No relevant statistical analysis involving comparisons between sample groups were made for this demonstration.
Data exclusions	No data was excluded from the research
Replication	All fixed cell datasets have two independent trials. Each trial contains 3 different cells. One cell from each condition was randomly chosen for displaying representative differences between different imaging conditions.<br>All IPSC cells used for phototoxicity and photobleaching tests have one experimental replicate, and each replicate contains 5 different cells per condition.<br>All replicates confirm the same results as stated in the manuscript.
Randomization	Cells were randomly allocated to experimental groups
Blinding	Because the data analysis for the comparisons used in this study require specific set up and post processing steps, blinding of experimental conditions was not possible.

Reporting for specific materials, systems and methods

We require information from authors about some types of materials, experimental systems and methods used in many studies. Here, indicate whether each material, system or method listed is relevant to your study. If you are not sure if a list item applies to your research, read the appropriate section before selecting a response.

Materials & experimental systems

n/a	Involved in the study
☒	☐ Antibodies
☐	☒ Eukaryotic cell lines
☒	☐ Palaeontology and archaeology
☒	☐ Animals and other organisms
☒	☐ Clinical data
☒	☐ Dual use research of concern

Methods

n/a	Involved in the study
☒	☐ ChIP-seq
☒	☐ Flow cytometry
☒	☐ MRI-based neuroimaging

Eukaryotic cell lines

Policy information about cell lines and Sex and Gender in Research

Cell line source(s)	RRID:CVCL_IR34 cells were purchased from the Coriell Institute for Medical Research, Cos7 (RRID:CVCL_0224) stably expressing Halo-H2b cells were a gift from Tim Brown at HHMI Janelia Research Campus
Authentication	All iPSC stem cell lines were authenticated by the Allen institute for cell science as indicated here: https:www.allencell.orgillustrated- overviews.html. Cos7-Halo-H2b cells were not authenticated.
Mycoplasma contamination	All iPSC stem cell lines were tested for mycoplasma prior to receipt by the Allen Institute for cell science. These cells were further tested for mycoplasma contamination via microscopy techniques. Cos7 Halo-H2b cells were tested for mycoplasma via microscopy techniques as well as isothermal PCR. There was no indication of mycoplasma contamination in any of the aforementioned tests.
Commonly misidentified lines (See ICLAC register)	No commonly misidentified cell lines were used in this study.
